# Bovine coronavirus enters PBIECs via membrane fusion and clathrin-, caveolin-mediated endocytosis, macropinocytosis

**DOI:** 10.1080/21505594.2026.2691344

**Published:** 2026-06-23

**Authors:** Chen Chen, Nannan Su, Long Zhao, Ning Li, Zhongyuan Ma, Bihao Luo, Wenhui Liu, Boli Song, Liang Zhang, Kangkang Guo

**Affiliations:** a College of Veterinary Medicine, Northwest A&F University, Yangling, Shaanxi, China; b Department of Medical Technology, Zhangzhou Health Vocational College, Zhangzhou, Fujian, China; c College of Animal Science and Technology, Xizang Vocational Technical College, Lhasa, Xizang, China

**Keywords:** Bovine coronavirus, membrane fusion, endocytic pathway, Rab proteins

## Abstract

Bovine coronavirus (BCoV) is an important pathogen that exhibits dual tropism for the respiratory and intestinal tracts, causing winter dysentery in adult cattle, diarrhea and respiratory infections in calves, thus imposing considerable economic losses on the global cattle industry. Our previous studies demonstrated that BCoV gains entry into susceptible HRT-18 cells through membrane fusion and clathrin-mediated endocytosis (CME). However, the precise mechanisms by which BCoV enters host cells remain incompletely elucidated, particularly in primary bovine intestinal epithelial cells (PBIECs). Importantly, as primary cells derived from the natural host, PBIECs more closely recapitulate the in *vivo* infection microenvironment than HRT-18 cell lines. In the present study, chemical inhibitors, RNA interference, and fluorescently labeled BCoV particles were used to define distinct entry pathways. Our data demonstrated that BCoV enters PBIECs via membrane fusion and three distinct endocytic pathways, including CME, caveolin-mediated endocytosis (CavME), and macropinocytosis. Dynamin, microtubules, cathepsins, and an acidic environment are essential for mediating endocytic entry, whereas cholesterol and TMPRSS2 are dispensable for this process. Furthermore, targeted interference with Rab5, Rab7, and Rab11 suppressed BCoV entry into PBIECs. Consistently, co-localization of fluorescently labeled BCoV with Rab5, Rab7, and Rab11 was observed by confocal microscopy, indicating that these Rab GTPases are involved in BCoV entry into PBIECs. These findings elucidate the entry mechanisms of BCoV and provide novel perspectives to enhance a more comprehensive understanding of the BCoV life cycle.

## Introduction

Bovine coronavirus (BCoV) is an enveloped, single-stranded positive-sense RNA virus belonging to the family *Coronaviridae*, genus *Betacoronavirus*. The genus also includes several highly pathogenic viruses, such as severe acute respiratory syndrome coronavirus 2 (SARS-CoV-2), human coronavirus OC43 (HCoV-OC43), and porcine hemagglutinating encephalomyelitis virus (PHEV) [1]. Notably, some *Beta*coronaviruses demonstrate a broad host range and a pronounced capacity for cross-species transmission, making the investigation of their entry mechanisms essential for understanding coronavirus pathogenesis and cross-species transmission mechanisms [2]. As a member of this genus, BCoV is a major pathogen responsible for substantial economic losses in the global cattle industry, and has also been reported to infect multiple ruminant species and swine [3]. Moreover, BCoV-like viruses have been detected in human samples, highlighting a non-negligible risk of interspecies transmission [3]. Like SARS-CoV-2, BCoV displays dual tropism for the respiratory and intestinal tracts, with intestinal infection causing epithelial cell damage and severe diarrhea [4]. Although the epidemiological and pathological characteristics of BCoV have been extensively investigated, the membrane fusion process involved in BCoV entry, endocytic entry routes, and endosomal trafficking remain poorly understood.

Viruses are obligate intracellular pathogens whose infectious cycles rely heavily on host cellular machinery [5]. Viral entry into host cells is an indispensable step for successful infection. Coronaviruses primarily utilize two distinct entry pathways:

plasma membrane fusion and endocytosis [6]. During plasma membrane fusion, the viral spike (S) protein is typically cleaved and activated at the cell surface, thereby triggering fusion between the viral envelope and the host plasma membrane and promoting rapid release of the nucleocapsid into the cytoplasm [7], as observed in SARS-CoV-2 and human coronavirus HKU1 infections [8]. In contrast, the endocytic pathway involves internalization of virions via membrane invagination, followed by further processing of the S protein within acidified endosomal compartments, ultimately leading to viral entry [8]. For example, Japanese encephalitis virus (JEV) enters human neuronal cells and rat neuroblastoma B104 through caveolae-mediated endocytosis (CavME) [9,10]; and the entry of porcine epidemic diarrhea virus (PEDV) into Vero cells and IPEC-J2 cells relies on clathrin-mediated endocytosis (CME) and CavME pathways [11]. Porcine deltacoronavirus (PDCoV) utilizes CavME to invade PK-15 cells [12], whereas entry into intestinal epithelial cells (IPI-2I) relies on CME and macropinocytosis [13]. Similarly, Newcastle disease virus (NDV) invades chicken macrophages through CavME [14], while entry into DF-1 relies on CME and macropinocytosis [15]. These studies indicate that a subset of viruses exploit the same entry pathway to infect different cell types, whereas others utilize distinct entry routes in different cells.

As an essential part of the small GTPase superfamily, Rab proteins play an important role in regulating intracellular vesicle transport, membrane fusion, and endosomal maturation [16]. Accumulating evidence indicates that many enveloped viruses hijack specific Rab proteins during host cell entry to ensure efficient transport of viral particles along the endocytic pathway and to facilitate subsequent membrane fusion events [17]. In the canonical endocytic pathway, Rab5 predominantly regulates the formation of endocytic vesicles as well as their fusion with early endosomes. Rab7 serves as a marker of late endosomes and lysosomes and mediates the maturation of early endosomes into late endosomes. In contrast, Rab11 functions in the recycling endosome pathway and controls the transport of recycled membrane proteins to the plasma membrane [16]. These Rab proteins not only regulate endosomal trafficking and maturation, but also critically influence whether viral particles can traffic to intracellular compartments permissive for membrane fusion and genome release [17]. Previous studies have shown that different viruses selectively exploit distinct Rab proteins depending on their entry strategies. For example, PHEV entry into mouse neuroblastoma (Neuro-2a) cells and influenza virus entry into HeLa cells require both Rab5 and Rab7 [18,19]; classical swine fever virus (CSFV) entry into PAMs depends on Rab5, Rab7, and Rab11 [20]; whereas dengue virus and West Nile virus entry into HeLa cells relies solely on Rab5 [21]. In summary, different viruses rely on distinct endosomal trafficking pathways to accomplish subsequent intracellular transport following entry into host cells. Our previous work revealed that BCoV enters susceptible HRT-18 cells through membrane fusion and the CME pathway, and requires both Rab7 and Rab11 [7]. However, the precise molecular mechanisms governing BCoV entry into PBIECs remain to be elucidated. The present work was designed to elucidate the entry pathways of BCoV into PBIECs and to define the roles of Rab proteins in this process. PBIECs isolated from neonatal calves were used as an *in vitro* model, and a combination of chemical inhibitors, RNA interference, and fluorescently labeled BCoV particles was employed to identify the host cellular factors involved in viral entry into PBIECs. In this study, we confirmed that BCoV enters PBIECs utilizing both membrane fusion and multiple endocytic pathways, including CME, CavME, and macropinocytosis. This entry process requires an acidic environment, dynamin, microtubules, and cathepsins, whereas TMPRSS2 and cholesterol are dispensable. Additionally, we identified Rab5, Rab7, and Rab11 as essential regulators involved in BCoV entry into PBIECs. Collectively, these findings provide deeper insight into the molecular mechanisms of BCoV entry and highlight potential host factors for the development of antiviral strategies.

## Results

### Isolation, identification of primary bovine intestinal epithelial cells (PBIECs) and BCoV infection

**Figure 1.**
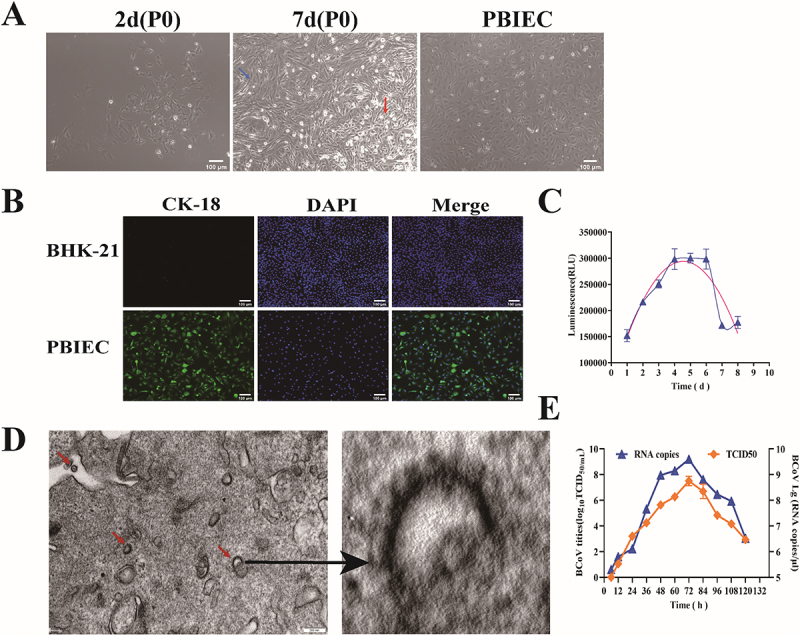
Isolation and characterization of PBIECs and BCoV infection (A) Isolation and purification of PBIECs. Blue arrows indicate fibroblast-like cells, and red arrows indicate epithelial-like cells. (B) identification of the epithelial origin of PBIECs by IFA detection of CK-18 expression. (C) growth curve of PBIECs. (D) TEM showing BCoV particles in infected PBIECs. Scale bar = 200 nm. (E) viral replication in PBIECs was analyzed by RT-qPCR and TCID_50_ assays following BCoV infection.

PBIECs were isolated from the ileum of neonatal calves. Briefly, ileal tissues were thoroughly washed with PBS containing antibiotics and then subjected to enzymatic digestion for cell isolation. PBIECs were maintained in DMEM/F12 medium containing various growth factors. As shown in Figure 1A, a limited number of cells adhered to the culture surface after 2 d. Following medium replacement and an additional 2 d of culture, two distinct cell morphologies were observed: cobblestone-like epithelial cells and spindle-shaped fibroblast-like cells. After 7 d of culture, cells were purified using a combination of differential adhesion and trypsinization. The purified cells were subsequently identified by immunofluorescence assay (IFA) using antibodies against the epithelial cell marker cytokeratin 18 (CK-18). The results confirmed that highly purified PBIECs were successfully obtained (Figure 1B), and purified PBIECs were used for subsequent research. The growth characteristics of purified PBIECs were evaluated with the CellTiter-Glo® 3D Cell Viability Assay to generate a growth curve. As shown in Figure 1C, PBIECs entered a plateau phase on day 4 of culture, which persisted for approximately 2 days, followed by a marked decrease in relative luminescence unit (RLU) values. These results indicate that PBIECs cultured in vitro undergo a period of growth arrest and subsequently enter cellular senescence. Transmission electron microscopy (TEM) revealed typical coronavirus particles in PBIECs following BCoV infection. The virions appeared spherical or pleomorphic, with diameters of approximately 80–120 nm, and displayed characteristic surface spike projections, indicating successful infection of PBIECs by BCoV (Figure 1D). Finally, the replication kinetics of BCoV in PBIECs were analyzed. Viral RNA loads and viral titers were determined by absolute quantitative PCR and the TCID_50_ assay, respectively, to generate viral growth curves. Our data showed that both intracellular BCoV RNA loads and viral titers reached their peak at 72 h post-infection (Figure 1E).

### BCoV entry into PBIECs depends on an acidic environment

**Figure 2.**
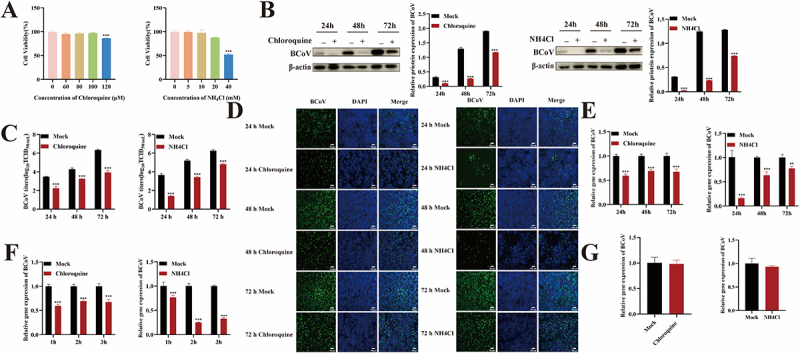
BCoV entry into PBIECs is dependent on an acidic environment. (A) the maximal non-cytotoxic concentrations of CQ and NH_4_Cl were determined by CCK-8 assay. (B) effects of CQ and NH_4_Cl treatment on N protein expression during BCoV infection were analyzed by Western blotting, with grayscale analysis shown as bar graphs. (C) effects of CQ and NH_4_Cl treatment on progeny virus titers were evaluated by TCID_50_ assay. (D) effects of CQ and NH_4_Cl treatment on the number of infected cells were assessed by IFA. Scale bar = 100 μm. (E) viral genomic copy numbers following CQ and NH_4_Cl treatment were quantified. (F, G) effects of CQ and NH_4_Cl treatment on BCoV entry and attachment were analyzed by RT-qPCR. Data are presented as the mean ± SD of three independent experiments (not significant, *p* > 0.05; **p < 0.01; ***p < 0.001).

Viral entry into many cell types relies on an acidic environment [22]. To determine whether BCoV entry into PBIECs is dependent on an acidic environment, chloroquine (CQ) or ammonium chloride (NH_4_Cl) were used as inhibitors of endosomal acidification. CCK-8 assays showed that the maximum non-cytotoxic concentrations of CQ and NH_4_Cl were 20 mM and 100 μM, respectively (Figure 2A). PBIECs were subjected to CQ or NH_4_Cl at their maximum nontoxic concentrations and subsequently infected with BCoV at an MOI of 1 for 24, 48, and 72 h, BCoV N protein expression was examined by Western blotting (Figure 2B), progeny viral titers were measured by the TCID_5__0_ assay (Figure 2C), the quantity of infected cells was measured by IFA (Figure 2D), and viral RNA loads were quantified by RT-qPCR (Figure 2E). These results showed that, compared with the untreated control group, both CQ and NH_4_Cl treatments significantly inhibited BCoV infection (*p* < 0.001). To additionally assess involvement of endosomal acidification in BCoV entry, PBIECs were cultured in the presence of CQ or NH_4_Cl Afterward, the cells were exposed to BCoV at an MOI of 5 for 1, 2, and 3 h. Experimental data showed that CQ and NH_4_Cl markedly impaired BCoV entry into cells (Figure 2F; *p* < 0.001). Conversely, after BCoV was incubated at 4 °C for 1 h, RT-qPCR analysis indicated that neither CQ nor NH_4_Cl impaired viral attachment. (Figure 2G; *p* > 0.05). These results indicate that BCoV entry into PBIECs depends on an acidic environment, whereas viral attachment does not.

### BCoV entry into PBIECs depends on a membrane fusion pathway

**Figure 3.**
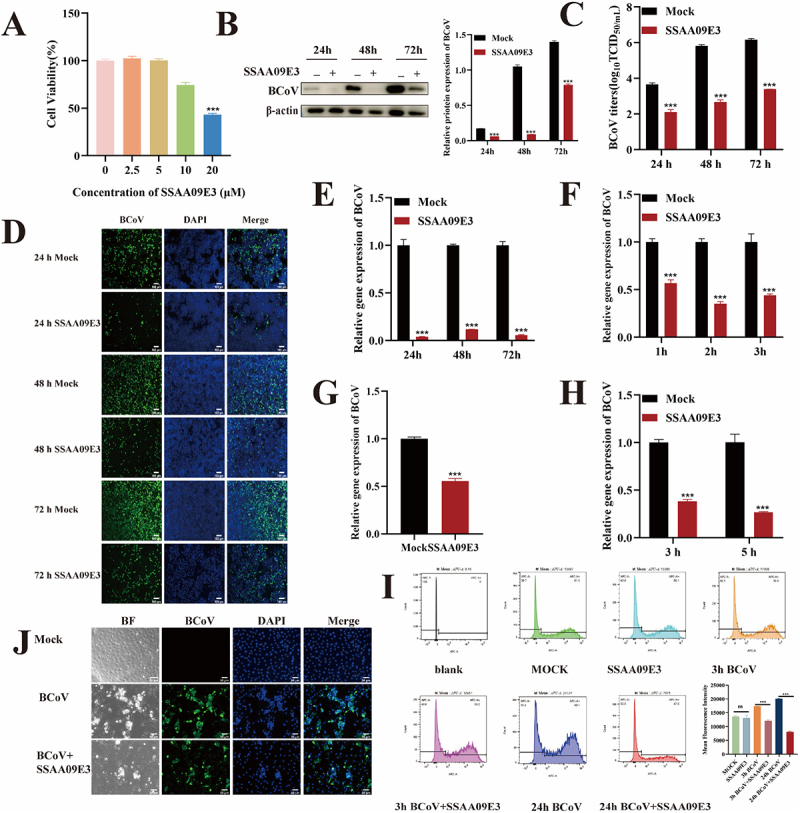
BCoV entry into PBIECs through membrane fusion. (A) the maximal non-cytotoxic concentration of SSAA09E3 was evaluated by CCK-8 assay. PBIECs were treated with SSAA09E3 for 24 h, BCoV infection was evaluated at 24, 48 and 72 h after infection by (B) Western blotting, (C) TCID50, (D) IFA, and (E) RT-qPCR. (F, G) effects of SSAA09E3 treatment on BCoV entry and attachment were analyzed by RT-qPCR. (H) effects of SSAA09E3 treatment on BCoV entry after viral attachment were investigated by RT-qPCR. (I) Representative histograms of DiD fluorescence in the APC channel and corresponding MFI analysis determined by flow cytometry. (J) effects of SSAA09E3 treatment on BCoV-induced syncytium formation. Scale bar = 50 μm. Data are presented as the mean ± SD of three independent experiments (not significant, *p* > 0.05; ***p < 0.001).

Coronaviruses generally enter host cells via membrane fusion and/or endocytic pathways. Given that BCoV belongs to the Coronaviridae, it is essential to assess the impact of membrane fusion on BCoV entry into PBIECs. In this study, SSAA09E3, a novel inhibitor of coronavirus membrane fusion, was used for subsequent experiments. First, CCK-8 assays were performed to determine the maximum non-cytotoxic concentration of SSAA09E3, which was confirmed to be 10 μM (Figure 3A). PBIECs were pretreated with SSAA09E3 at 10 μM for 24 h and subsequently infected with BCoV (MOI = 1). At 24, 48, and 72 h post-infection, BCoV N protein expression was detected by Western blotting (Figure 3B; *p* < 0.001), progeny virus titers were measured via the TCID_50_ assay (Figure 3C; *p* < 0.001), the number of infected cells was quantified using IFA (Figure 3D), and viral genome copy numbers were determined by RT-qPCR (Figure 3E; *p* < 0.001). These results showed that SSAA09E3 significantly inhibited BCoV infection, indicating that BCoV infection of PBIECs depends on membrane fusion. Subsequently, the effect of SSAA09E3 on BCoV attachment and entry into PBIECs was evaluated, demonstrating that SSAA09E3 strongly suppressed the entry and attachment (*p* < 0.001; Figure 3F,G). To verify whether the effect of SSAA09E3 on BCoV entry was mediated by inhibiting viral attachment, PBIECs were first incubated with BCoV at 4 °C to allow attachment. After removal of unattached viral particles, cells were treated with SSAA09E3 for 3 or 5 h. SSAA09E3 administration for 3 and 5 h significantly reduced viral RNA levels (Figure 3H; *p* < 0.001), indicating that SSAA09E3 inhibits BCoV entry independently of viral attachment. To provide additional evidence of the inhibitory effect of SSAA09E3 on BCoV-mediated membrane fusion, cell membranes were labeled with 1,1’-dioctadecyl-3,3,3’,3’-tetramethylindodicarbocyanine (DiD), and mean fluorescence intensity (MFI) was measured by flow cytometry. Increased membrane fusion and syncytium formation correlate with elevated MFI values. In uninfected groups (Mock and SSAA09E3 alone),

no significant differences in MFI were observed between groups, indicating that SSAA09E3 does not affect cell-cell fusion in the absence of viral infection (*p* > 0.05; Figure 3I). In contrast, MFI in the Mock, 3 h BCoV, and 48 h BCoV groups increased significantly with prolonged BCoV infection, demonstrating that BCoV infection induces membrane fusion in a time-dependent manner (*p* < 0.001; Figure 3I). Notably, in both the 3 h and 48 h infection groups, SSAA09E3 led to a significant reduction in MFI relative to the corresponding untreated BCoV-infected groups, indicating that SSAA09E3 effectively inhibits BCoV-induced membrane fusion (Figure 3I; *p* < 0.001). To directly visualize BCoV-induced syncytium formation, PBIECs were examined using fluorescence microscopy. Consistent with the flow cytometry results, BCoV infection induced prominent syncytium formation, which was markedly suppressed by SSAA09E3 treatment (Figure 3J). Collectively, these findings demonstrate that the entry of BCoV into PBIECs relies on a membrane fusion pathway.

### Roles of cathepsins and TMPRSS2 in BCoV entry into PBIECs

**Figure 4.**
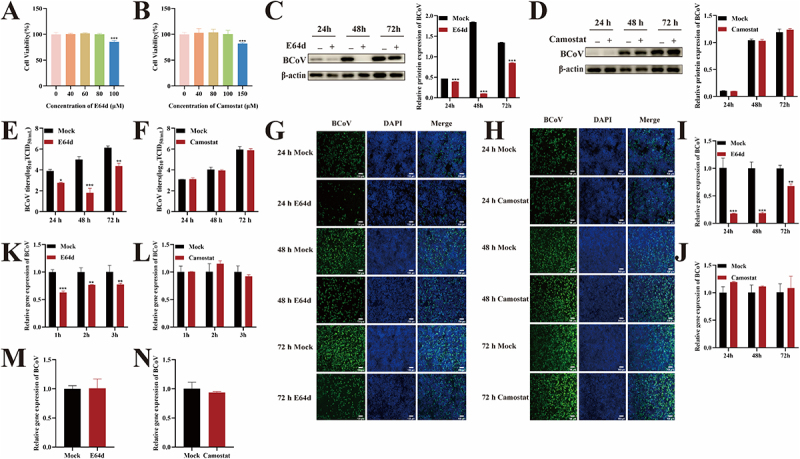
Roles of cathepsins and TMPRSS2 in BCoV entry into PBIECs. (A, B) the maximal non-cytotoxic concentration of E64D and camostat were determined by CCK-8 assay. (C, D) impacts of E64D and camostat treatment on BCoV N protein expression were evaluated by Western blotting, with densitometric quantification shown as bar graphs. (E, F) impacts of E64D and camostat treatment on progeny virus titers were determined by TCID_50_ assay. (G, H) effects of E64D and camostat treatment on the number of infected cells were assessed by IFA. Scale bar = 100 μm. (I, J) effects of E64D and camostat treatment on viral genomic copy numbers were analyzed. (K, L) effects of E64D and camostat treatment on BCoV entry were analyzed. (M, N) effects of E64D and camostat treatment on BCoV attachment were analyzed by RT-qPCR. Data are presented as the mean ± SD of three independent experiments (not significant, *p* > 0.05; *p < 0.05; **p < 0.01; ***p < 0.001).

Cathepsins and TMPRSS2 are two major classes of host proteases that play pivotal roles in viral entry, particularly for enveloped viruses such as coronaviruses. These proteases facilitate viral entry through distinct molecular mechanisms [23]. To explore the roles of cathepsins and TMPRSS2 in BCoV entry into PBIECs, we used the cathepsin inhibitor E64d and the TMPRSS2 inhibitor camostat to selectively inhibit their enzymatic activities. Based on CCK-8 assays, 80 μM (Figure 4A) and 100 μM (Figure 4B) were identified as the maximum non-cytotoxic concentrations for E64d and camostat, respectively. PBIECs were preincubated with E64d or camostat, after which the effects of these inhibitors on BCoV infection, entry, and attachment were evaluated. Compared with untreated controls, E64d treatment significantly reduced BCoV infection, as reflected by decreases in multiple infection-related parameters (Figure 4C–G, and I; *p* < 0.05), and markedly inhibited viral entry into PBIECs (Figure 4K; *p* < 0.01), while viral attachment remained unaffected (Figure 4M, *p* > 0.05). In contrast, camostat treatment had no detectable effect on BCoV infection (*p* > 0.05; Figure 4D–J), entry or attachment (*p* > 0.05; Figure 4L,N). These results indicate that cathepsins are required for efficient BCoV entry into PBIECs, whereas TMPRSS2 is not essential for this process.

### BCoV enters PBIECs via clathrin-mediated endocytosis

**Figure 5.**
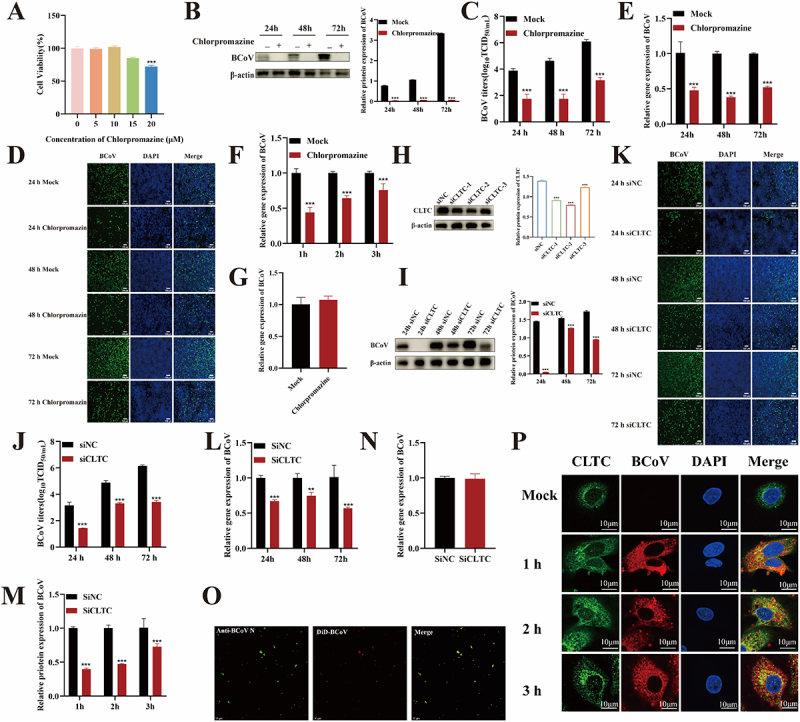
BCoV enters PBIECs via CME. (A) the non-cytotoxic concentration of CPZ was validated by CCK-8 assay. (B) CPZ treatment reduced BCoV N protein level as determined by Western blotting, with grayscale analysis shown as bar graphs. (C) CPZ treatment reduced virus titers, as measured by the TCID_50_ assay. (D) CPZ treatment decreased the proportion of infected cells as determined by IFA. Scale bar = 100 μm. (E) CPZ treatment reduced viral genomic copy numbers as determined by RT-qPCR. (F) CPZ treatment inhibited BCoV entry as determined by RT-qPCR. (G) CPZ treatment had no significant effect on BCoV attachment as determined by RT-qPCR. (H) screening of siRnas targeting CLTC, and the most efficient siRNA was selected. (I-L). Western blotting (I), TCID_50_ assay (J), IFA (K), and RT-qPCR (L) were performed to assess the effects of CLTC silencing on BCoV infection (M, N) the impact of CLTC silencing on BCoV entry and attachment were analyzed by RT-qPCR. (O) detection of DiD-labeled and antibody-stained BCoV particles. (P) confocal microscopy analysis of the colocalization of DiD-labeled BCoV with CLTC during viral entry. Scale bar = 10 μm. Data are presented as the mean ± SD of three independent experiments (not significant, *p* > 0.05; **p < 0.01; ***p < 0.001).

The clathrin-mediated endocytosis (CME) pathway is commonly employed by most coronaviruses to gain entry into host cells [24]. To evaluate the role of CME in the entry of BCoV into PBIECs, chlorpromazine (CPZ), a well-known CME inhibitor, was employed. PBIECs were treated with the maximum non-cytotoxic concentration of CPZ (15 μM, Figure 5A) and infected with BCoV for 24, 48 and 72 h. Western blotting (Figure 5B), IFA (Figure 5C), TCID_50_ assays (Figure 5D), and RT-qPCR (Figure 5E) showed that CPZ treatment markedly decreased N protein expression, progeny virus titers, the number of infected cells, and viral RNA copy numbers. These results indicate that CPZ treatment significantly suppresses BCoV infection (*p* < 0.001). To further determine whether CME is implicated in the initial stage of BCoV entry, PBIECs were preincubated with CPZ prior to BCoV infection. RT-qPCR assays showed that CPZ treatment significantly inhibited BCoV entry (Figure 5F; *p* < 0.001). In contrast, when CPZ-treated PBIECs were incubated with BCoV at 4 °C to permit viral attachment, CPZ treatment had no impact on viral attachment (Figure 5G; *p* > 0.05). To further validate these findings, siRNAs targeting clathrin heavy chain (CLTC) were designed and synthesized. Among them, CLTC-3 displayed robust silencing effects, and was therefore selected for further experimental studies (Figure 5H). Silencing clathrin expression significantly inhibited BCoV infection and entry into PBIECs (Figure 5I–M; *p* < 0.01), while viral attachment remained unaffected (Figure 5N; *p* > 0.05). To validate the efficiency of DiD labeling, BCoV particles were labeled with DiD and analyzed via confocal microscopy for colocalization with FITC-labeled viral particles (Figure 5O). The strong colocalization confirmed the high efficiency of DiD labeling, supporting its suitability for subsequent experiments. Confocal microscopy observed clear colocalization between DiD-labeled BCoV and clathrin during the early stage of viral entry (Figure 5P). Taken together, these data demonstrate that BCoV enters PBIECs via CME, whereas viral attachment to PBIECs occurs independently of this pathway.

### Dynamin is essential for BCoV entry into PBIECs

**Figure 6.**
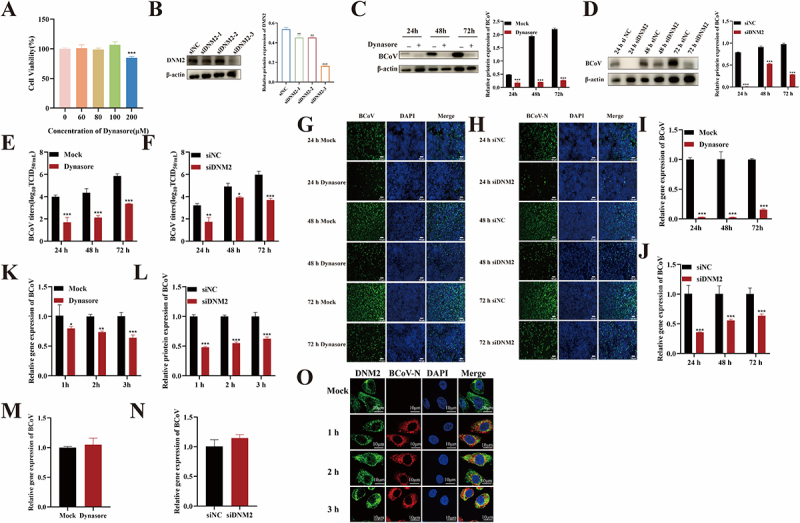
Dynamin is implicated in BCoV entry into PBIECs. (A) the maximal non-cytotoxic concentration of dynasore was determined via the CCK-8 assay. (B) screening of siRnas targeting dynamin 2 (DNM2) to identify the most efficient siRNA. (C, D) effects of dynasore treatment and DNM2 silencing on BCoV N protein expression were analyzed by Western blotting, with grayscale analysis shown as bar graphs. (E, F) effects of dynasore treatment and DNM2 silencing on progeny virus titers were analyzed by TCID_50_ assay. (G, H) effects of dynasore treatment and DNM2 silencing on the proportion of infected cells were assessed by IFA. Scale bar = 100 μm. (I, J) effects of dynasore treatment and DNM2 silencing on viral genomic copy numbers were analyzed by RT-qPCR. (K, L) effects of dynasore treatment and DNM2 silencing on viral genomic copy numbers during the entry stage were analyzed by RT-qPCR. (M, N) effects of dynasore treatment and DNM2 silencing on viral genomic copy numbers during the attachment stage were analyzed. (O) confocal microscopy analysis of the colocalization of DiD-labeled BCoV with dynamin. Scale bar = 10 μm. Data are presented as the mean ± SD of three independent experiments (not significant, *p* > 0.05; *p < 0.05; **p < 0.01; ***p < 0.001).

The entry of many viruses depends on dynamin-mediated vesicle fission [25]. To assess dynamin’s contribution to BCoV entry into PBIECs, we used the specific inhibitor dynasore and dynamin (DNM)-targeted siRNA. The maximum non-cytotoxic concentration of dynasore was 100 μM (Figure 6A). Western blotting showed that siDNM2 had the highest knockdown efficiency (Figure 6B). PBIECs were treated with dynasore (100 μM) or transfected with siDNM2 and subsequently infected with BCoV at an MOI of 1. Results from Western blotting, TCID_50_ assays, IFA, and RT-qPCR assays revealed that both dynasore treatment and dynamin silencing substantially suppressed BCoV infection compared with the control groups, suggesting that dynamin is involved in BCoV infection (*p* < 0.05; Figure 6C–J). To further distinguish the role of dynamin in viral entry vs attachment, PBIECs treated with dynasore or dynamin-targeted siRNA were examined for BCoV entry and attachment. RT-qPCR analysis demonstrated that both dynasore treatment and dynamin silencing markedly suppressed BCoV entry (*p* < 0.05; Figure 6K,L), whereas viral attachment was not affected (*p* > 0.05; Figure 6M,N). Moreover, confocal microscopy revealed clear colocalization of dynamin with DiD-labeled BCoV particles at 1, 2, and 3 h post-infection (Figure 6O). Collectively, these data demonstrate that dynamin is essential for BCoV entry into PBIECs.

### BCoV entry into PBIECs depends on the caveolin-mediated endocytic pathway

**Figure 7.**
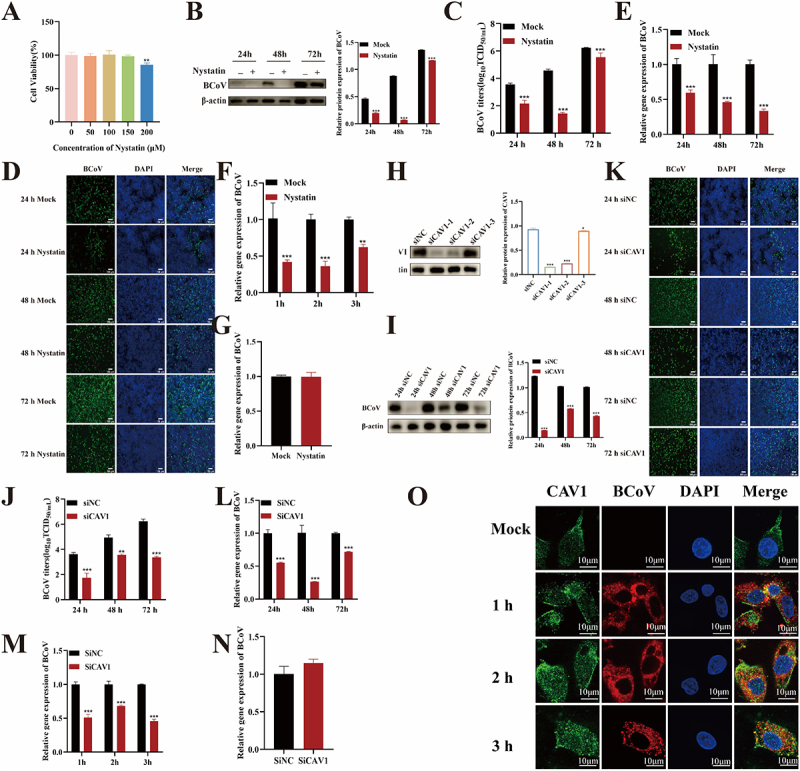
BCoV enters PBIECs via CavME. (A) the maximal non-cytotoxic concentration of nystatin was established via CCK-8 assay. (B) effects of nystatin treatment on BCoV N protein expression were probed via Western blotting, with grayscale analysis shown as bar graphs. (C) effects of nystatin treatment on BCoV titers were analyzed by TCID_50_ assay. (D) IFA was used to assess the effects of nystatin on BCoV-infected cells. Scale bar = 100 μm. (E-G) effects of nystatin treatment on viral infection, entry, and attachment were analyzed by RT-qPCR. (H) screening of siRnas targeting CAV1 to determine silencing efficiency. (I-L) the impacts of CAV1 silencing on BCoV infection in PBIECs were analyzed by Western blotting (I), TCID_5__0_ assay (J), IFA (K), and RT-qPCR (L). (M, N) effects of CAV1 silencing on BCoV entry and attachment were analyzed by RT-qPCR. (O) confocal microscopy analysis of the colocalization of DiD-labeled BCoV with CAV1 at 1, 2, and 3 h post-infection. Scale bar = 10 μm. Data are presented as the mean ± SD of three independent experiments (not significant, *p* > 0.05; **p < 0.01; ***p < 0.001).

Caveolin-mediated endocytosis (CavME) serves as a key cellular internalization route that relies on specialized membrane invaginations formed by caveolin proteins [26]. The caveolae-disrupting agent nystatin was used to investigate the role of CavME in BCoV entry. PBIECs were pretreated with nystatin (150 μM, Figure 7A) and cell samples were collected after infection with BCoV. Subsequent analyses revealed that nystatin treatment markedly reduced BCoV N protein expression (Figure 7B; *p* < 0.001), progeny virus titers (Figure 7C), *p* < 0.001), infected cell numbers (Figure 7D), and viral RNA copy numbers (*p* < 0.001; Figure 7E), indicating that efficient BCoV infection relies on the caveolin-mediated endocytic pathway. To further substantiate these observations, siRNAs targeting Caveolin-1 (CAV1) were designed and evaluated, and the siRNA with the best knockdown efficiency was selected (Figure 7H). Silencing of CAV1 expression resulted in a substantial attenuation of BCoV infection in PBIECs, as demonstrated by multiple infection-related readouts (Figure 7I–L; *p* < 0.01), further supporting the involvement of caveolin in BCoV infection. The specific role of CavME in viral entry and attachment was then examined. PBIECs pretreated with nystatin were infected with BCoV (MOI = 5). The results demonstrated that nystatin treatment inhibited BCoV entry (Figure 7F; *p* < 0.01) without affecting viral attachment (Figure 7G; *p* > 0.05). Consistently, silencing of CAV1 by siRNA also suppressed BCoV entry (Figure 7M; *p* < 0.05) without exerting any detectable impact on viral attachment (Figure 7N; *p* > 0.05). Finally, at 1, 2, and 3 h post-infection, clear colocalization between caveolin and DiD-labeled BCoV particles was observed in PBIECs (Figure 7O). Collectively, these results demonstrate that efficient BCoV entry into PBIECs relies on CavME.

### BCoV enters PBIECs via the macropinocytosis pathway

In addition to the classical CME and CavME, macropinocytosis represents another important pathway for viral internalization. To determine whether macropinocytosis facilitates BCoV entry into PBIECs, blebbistatin was employed to block this pathway.

**Figure 8.**
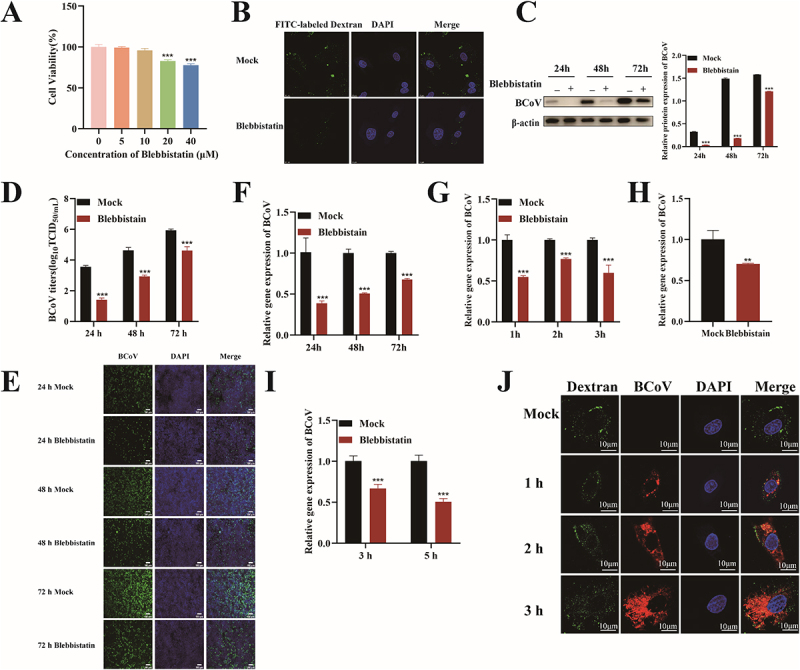
BCoV entry into PBIECs involves macropinocytosis. (A) the maximal non-cytotoxic concentration of blebbistatin was determined by CCK-8 assay. PBIECs were treated with blebbistatin for 24 h and then infected with BCoV. (B) 70-kDa FITC-labeled dextran uptake assay confirmed the suppression of micropinocytosis by blebbistatin in PBIECs. Scale bar = 10 μm. Viral infection was assessed by (C) Western blotting, with grayscale analysis shown as bar graphs (D) TCID_50_ assay, (E) IFA, and(F) RT-qPCR. Scale bar = 100 μm. (G, H) effects of blebbistatin treatment on BCoV entry and attachment were analyzed by RT-qPCR. (I) effects of blebbistatin treatment on BCoV entry after viral attachment were analyzed by RT-qPCR. (J) colocalization of DiD-BCoV and FITC-labeled dextran verifies BCoV entry via macropinocytosis in PBIECs. Scale bar = 10 μm. Data are presented as the mean ± SD of three independent experiments (not significant, *p* > 0.05; ***p < 0.001).

First, the maximum non-cytotoxic concentration of blebbistatin was 10 μM (Figure 8A). The FITC-labeled dextran is a specific marker of macropinocytosis and is internalized via macropinocytic vesicles rather than CME or CavME. PBIECs were pretreated with 10 μM blebbistatin for 24 h, followed by FITC-labeled dextran incubation to assess macropinocytic activity. Confocal microscopy showed that FITC-labeled dextran fluorescence intensity was markedly decreased in blebbistatin-treated cells relative to the control cells (Figure 8B), confirming that blebbistatin effectively inhibited the macropinocytic pathway in this experimental system. The effect of blebbistatin (10 μM) on BCoV infection in PBIECs was analyzed by Western blotting (Figure 8C), TCID_5__0_ assays (Figure 8D), IFA (Figure 8E), and RT-qPCR (Figure 8F). Compared with the untreated control group, blebbistatin treatment markedly decreased BCoV infection (*p* < 0.001), indicating that BCoV infection of PBIECs is closely associated with the macropinocytosis pathway. Subsequently, the role of blebbistatin in viral entry was further examined. The results showed that blebbistatin treatment significantly reduced intracellular BCoV RNA levels during the entry stage (Figure 8G; *p* < 0.001). Next, the impact of blebbistatin on viral attachment was evaluated. RT-qPCR results indicated that blebbistatin pretreatment reduced BCoV attachment to PBIECs (Figure 8H; *p* < 0.01). To clarify whether the observed inhibition of viral entry was merely a consequence of impaired attachment, BCoV was first allowed to bind to PBIECs, after which blebbistatin was added. Under these conditions, blebbistatin treatment for 3 h or 5 h still significantly suppressed BCoV infection (Figure 8I; *p* < 0.001), demonstrating that blebbistatin inhibits BCoV entry independently of its effect on viral attachment. Finally, at 1, 2, and 3 h post-infection, clear colocalization between FITC-labeled dextran and DiD-labeled BCoV particles was observed in PBIECs (Figure 8J). Collectively, these data strongly demonstrate that BCoV entry into PBIECs is mediated via the macropinocytosis pathway.

### Cholesterol is dispensable for BCoV entry into PBIECs

**Figure 9.**
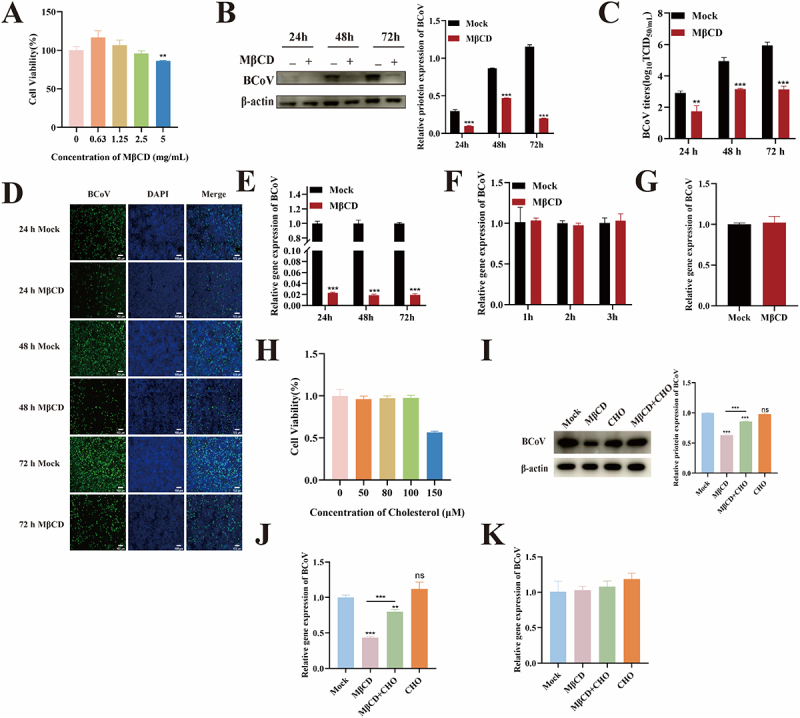
Cholesterol is dispensable for BCoV entry into PBIECs. (A) based on results from the CCK-8 assay, the non-cytotoxic concentration of MβCD was identified (B) effects of MβCD treatment on BCoV N protein expression were analyzed by Western blotting. (C) effects of MβCD treatment on progeny virus titers were analyzed by TCID_50_ assay. (D) effects of MβCD treatment on the number of infected cells were assessed by IFA. Scale bar = 100 μm. (E) the effect of MβCD treatment on total viral genome copy numbers was quantified via RT-qPCR. (F, G) RT-qPCR was utilized to interrogate the impacts of MβCD treatment on BCoV entry and attachment. (H) the maximal non-cytotoxic concentration of cholesterol was determined by CCK-8 assay. (I) Western blot analysis was used to evaluate BCoV N protein expression levels, and densitometric analysis was performed and presented as a bar graph. (J) RT-qPCR was performed to assess BCoV viral RNA copy numbers. (K) RT-qPCR was used to evaluate the effect of cholesterol on viral entry. Data are presented as the mean ± SD of three independent experiments (not significant, *p* > 0.05; *p < 0.05; **p < 0.01; ***p < 0.001).

Cholesterol plays a pivotal role in the viral life cycle, and depletion of cellular cholesterol is known to impair viral entry [27]. Methyl-β-cyclodextrin (MβCD) at 2.5 mg/mL (Figure 9A) was used to elucidate the contribution of cholesterol to BCoV infection of PBIECs. The results showed that MβCD treatment markedly reduced BCoV infection, as demonstrated by decreased viral N protein expression, progeny virus titers, numbers of infected cells, and viral RNA copy numbers (*p* < 0.05; Figure 9B–E), indicating that cholesterol is involved in BCoV infection of PBIECs. To further delineate the stage at which cholesterol is required, the effects of cholesterol depletion on viral entry and attachment were examined (Figures 9F,G). The data revealed that cholesterol depletion did not inhibit BCoV attachment or entry (*p* > 0.05).

To further clarify whether cholesterol is involved in BCoV entry or infection, we performed additional cholesterol depletion and replenishment experiments. PBIECs were divided into four groups: an untreated control group (Mock), an MβCD-treated group, an MβCD + cholesterol replenishment group, and a cholesterol-only group. Prior to these experiments, CCK-8 assays identified 100 μM as the maximum non-cytotoxic concentration of cholesterol for PBIECs (Figure 9H). Following the treatments described above, PBIECs were infected with BCoV at an MOI of 1. Cell samples were collected at 48 h post-infection, and viral infection was evaluated by Western blotting and RT-qPCR. The results showed that MβCD treatment significantly reduced BCoV N protein expression and viral RNA copy numbers (Figure 9I, J ;*p* < 0.001). Importantly, cholesterol replenishment after MβCD treatment effectively restored BCoV N protein expression and viral RNA copy numbers (*p* < 0.001). In contrast, cholesterol-only treatment exerted no significant effects on BCoV N protein expression or viral RNA copy numbers compared with the control group (*p* > 0.05). These findings indicate that cholesterol replenishment can partially rescue BCoV infection following cholesterol depletion. To explore the role of cholesterol in BCoV entry, PBIECs were harvested at 2 h post-infection after the above treatments. RT-qPCR analysis demonstrated that neither cholesterol depletion by MβCD, cholesterol replenishment after MβCD treatment, nor cholesterol-only treatment caused significant changes in viral RNA copy numbers during viral entry (Figure 9K; *p* > 0.05). Altogether, these observations demonstrate that cholesterol is not essential for BCoV attachment or entry into PBIECs.

### Microtubules are involved in BCoV entry into PBIECs

**Figure 10.**
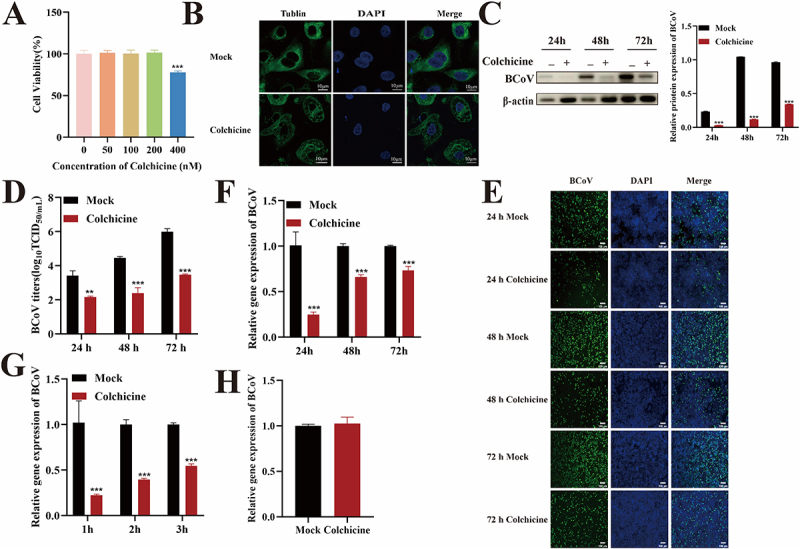
Microtubules are required for BCoV entry into PBIECs. (A) the maximal non-cytotoxic concentration of colchicine was determined by CCK-8 assay. (B) effects of colchicine treatment at the maximal non-cytotoxic concentration on microtubule organization were examined by confocal microscopy. PBIECs were treated with colchicine, BCoV infection was evaluated by (C) Western blotting, (D) TCID_50_ assay, (E) IFA, and (F) RT-qPCR. (G, H) RT-qPCR analysis was utilized to assess the impacts of colchicine treatment on BCoV entry and attachment. Scale bar = 100 μm. Data are presented as the mean ± SD of three independent experiments (not significant, *p* > 0.05; **p < 0.01; ***p < 0.001).

During viral entry into host cells, microtubules provide important structural support and intracellular transport routes [28]. Colchicine disrupts microtubule dynamics by inhibiting microtubule polymerization and promoting the depolymerization of preexisting microtubules, thereby dismantling the cellular microtubule network. To investigate the functional role of microtubules in BCoV entry into PBIECs, we employed colchicine as a specific pharmacological inhibitor. Initially, CCK-8 assays were performed to establish the maximum non-cytotoxic concentration of colchicine, which was identified as 200 nM (Figure 10A). PBIECs were then treated with colchicine at this concentration, and confocal microscopy revealed that the normally dense microtubule network became markedly dispersed, confirming effective microtubule depolymerization at the selected concentration (Figure 10B). Subsequently, PBIECs were preincubated with colchicine and infected with BCoV at an MOI of 1. We then analyzed viral N protein expression, infected cell numbers, progeny virus titers, and viral RNA copy levels. All of these parameters were significantly decreased in colchicine-treated cells (Figure 10C–F; *p* < 0.01), indicating that an intact microtubule network is essential for efficient BCoV infection of PBIECs. To further define the specific stage where microtubules act during BCoV infection, we separately evaluated viral attachment and entry. Our findings revealed that colchicine treatment suppressed viral entry (Figure 10G; *p* < 0.001) without impairing the attachment stage (Figure 10H; *p* > 0.05). Taken together, these observations suggest that microtubules contribute to BCoV entry into PBIECs.

### Visualization of BCoV entry into PBIECs

**Figure 11.**
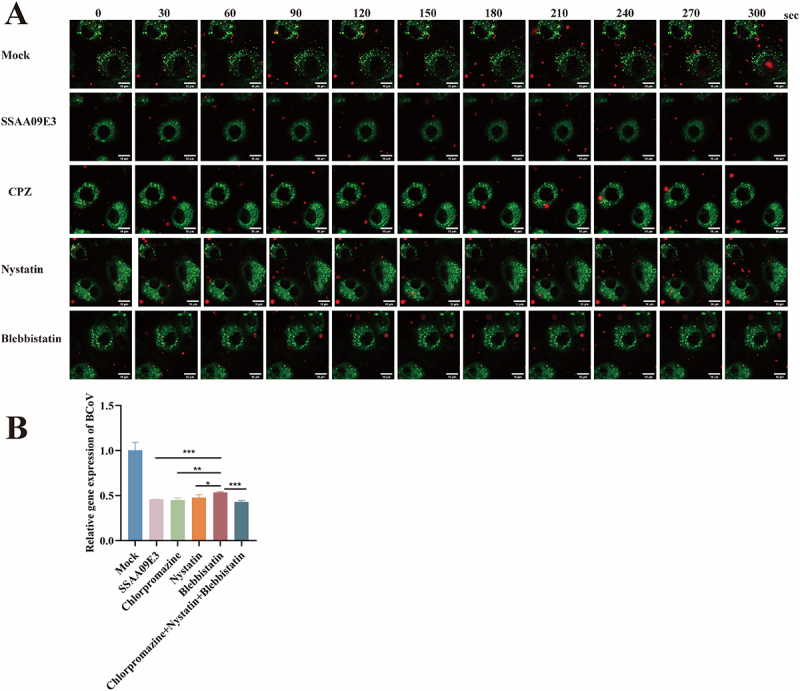
Visualization and quantitative analysis of BCoV entry into PBIECs following inhibitor treatment (A) Visualization of BCoV entry into PBIECs using DiD-labeled virus and DiO membrane staining. Scale bar = 10 μm. (B) RT-qPCR analysis of the effects of different inhibitors on BCoV entry efficiency. Data are presented as the mean ± SD of three independent experiments (not significant, *p* > 0.05; *p < 0.05; **p < 0.01; ***p < 0.001).

To directly visualize the entry process of BCoV and further verify the roles of membrane fusion, CME, CavME, and macropinocytosis in BCoV entry into PBIECs, we performed a DiD – DiO dual-labeling assay combined with high-speed super-resolution confocal microscopy. Five experimental groups were set up to assess distinct viral entry pathways: a control group, a CPZ-treated group (for CME inhibition), a nystatin-treated group (for CavME inhibition), a blebbistatin-treated group (for macropinocytosis inhibition), and an SSAA09E3-treated group (for membrane fusion inhibition). PBIECs were pre-treated with corresponding pathway inhibitors, followed by DiO labeling of cell membranes and DiD labeling of BCoV particles using the lipophilic membrane dye DiD. DiD-labeled BCoV was incubated with DiO-labeled PBIECs at 4°C for 1 h to enable stable viral attachment to the cell surface. The viral entry process was then monitored in real time over a 5-min period. High-speed super-resolution confocal laser scanning microscopy revealed that in the control group, most DiD-labeled BCoV particles penetrated the DiO-labeled plasma membrane and entered the cytoplasm (Figure 11A). In contrast, in all inhibitor-treated groups, the majority of viral particles remained adherent to the cell surface and failed to internalize (Figure 11A). Collectively, these results demonstrate that chlorpromazine, nystatin, blebbistatin, and SSAA09E3 effectively inhibit BCoV entry into host cells, further confirming the involvement of membrane fusion, CME, CavME, and macropinocytosis in the BCoV entry process.

To further clarify the relationship among the multiple pathways involved in BCoV entry into PBIECs, cells were pretreated for 24 h with SSAA09E3, chlorpromazine, nystatin, blebbistatin, or a combination of chlorpromazine, nystatin, and blebbistatin. Viral entry efficiency was then evaluated at 2 h post-entry by RT-qPCR analysis. The results showed that inhibition of any single pathway reduced viral entry efficiency to approximately 46%–59% (Figure 11B). Slight differences in the contribution of the four pathways were observed, with inhibition of macropinocytosis producing a significantly weaker inhibitory effect than inhibition of the other pathways (*p* < 0.05), whereas no significant differences were detected among the membrane fusion, CME, and CavME groups (*p* > 0.05). These findings indicate that macropinocytosis plays a comparatively minor role in BCoV entry. Importantly, inhibition of a single pathway failed to completely block viral entry, and simultaneous inhibition of the three endocytic pathways did not produce an additive inhibitory effect. Taken together, these results indicate that membrane fusion, CME, CavME, and macropinocytosis function in parallel during BCoV entry into PBIECs.

### Dependence of BCoV entry on endosomes

**Figure 12.**
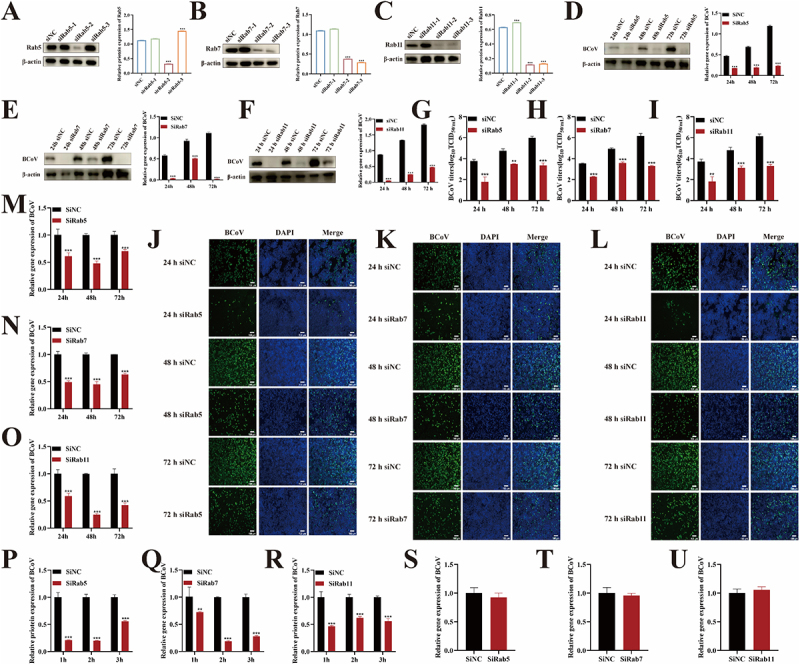
Rab proteins contribute to the entry of BCoV into PBIECs. (A-C) screening of siRnas targeting Rab5, Rab7, and Rab11 to evaluate silencing efficiency. (D-F) effects of Rab5, Rab7, and Rab11 silencing on BCoV N protein expression were assayed by Western blotting, with densitometric quantification shown as bar graphs. (G-I) effects of Rab5, Rab7, and Rab11 silencing on progeny virus titers were analyzed by TCID_50_ assay. (J-L) effects of Rab5, Rab7, and Rab11 silencing on the number of infected cells were assessed by IFA. Scale bar = 100 μm. (M-O) effects of Rab5, Rab7, and Rab11 silencing on viral genomic copy numbers were assayed by RT-qPCR. (P-U) effects of Rab5, Rab7, and Rab11 silencing on viral genomic copy numbers during the entry and attachment stages were assayed utilizing RT-qPCR. Data are presented as the mean ± SD of three independent experiments (not significant, *p* > 0.05; **p < 0.01; ***p < 0.001).

**Figure 13.**
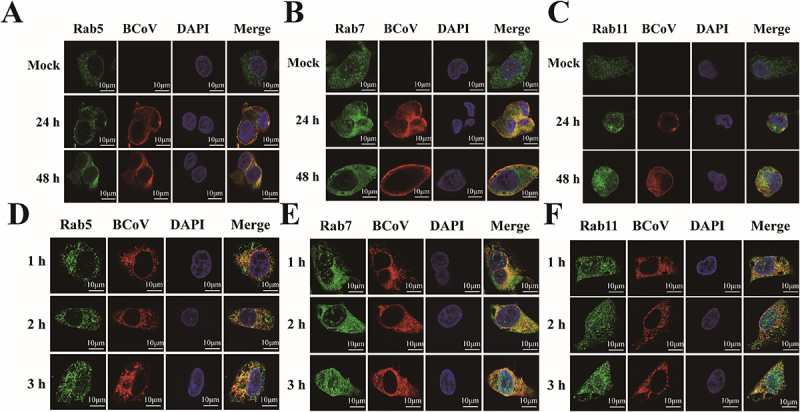
Colocalization of BCoV with Rab5, Rab7, and Rab11 during infection and entry. (A-C) colocalization of BCoV with Rab5, Rab7, and Rab11 at 24 and 48 h post-infection. (D-F) colocalization of DiD-labeled BCoV with Rab5, Rab7, and Rab11 at 1, 2, and 3 h post-infection. Scale bar = 10 μm.

Rab proteins constitute a major branch of the small Ras-related GTPase superfamily in eukaryotic cells and play key roles in the coordination of intracellular membrane transport processes [24]. Multiple viruses exploit Rab-regulated endosomal pathways to facilitate their entry and subsequent intracellular transport. Early endosomes (Rab5), late endosomes (Rab7), and recycling endosomes (Rab11) are therefore frequently investigated in studies of virus entry. In this study, the participation of Rab5-, Rab7-, and Rab11-associated endosomal pathways in BCoV entry into PBIECs was examined by targeted siRNA-mediated silencing. The silencing efficiencies of siRNAs against three Rabs were validated by Western blotting, and the most effective siRNAs were selected for subsequent experiments (Figure 12A–C). Following transfection with siRabs, PBIECs were infected with BCoV, and viral N protein expression, infected cell numbers, progeny virus titers, and viral RNA copy numbers were assessed. Silencing of Rab5, Rab7, or Rab11 markedly reduced BCoV infection in PBIECs (Figure 12D–O; *p* < 0.01). The significance of Rab proteins for BCoV entry was further analyzed. Quantification of viral RNA levels demonstrated that silencing Rab5, Rab7, or Rab11 significantly impaired BCoV entry into PBIECs (Figure 12P–R; *p* < 0.01), whereas viral attachment was not affected (Figure 12S–U; *p* > 0.05). To visualize the intracellular trafficking of BCoV, confocal microscopy was performed. At 24 and 48 h post-infection, BCoV particles exhibited clear colocalization with Rab5-, Rab7-, and Rab11-positive compartments (Figure 13A–C). Consistently, during the early entry phase (1, 2, and 3 h post-infection), Rab5, Rab7, and Rab11 were also found to colocalize with DiD-labeled BCoV in our observations (Figure 13D–F). Collectively, these data reveal that BCoV entry into PBIECs requires the coordinated involvement of early, late, and recycling endosomes.

## Discussion

Efficient viral entry is a fundamental prerequisite for viral transmission. For enveloped viruses, entry not only depends on the interaction between viral proteins and host cell surface receptors and the subsequent fusion of the viral envelope with the host cell membrane mediated by specialized fusion proteins, but also relies on endocytic pathways, which have been extensively studied and are considered essential for viral entry [29]. BCoV causes lung and intestinal diseases, leading to substantial economic losses in animal husbandry. Nevertheless, the mechanisms governing BCoV entry remain incompletely characterized. In our previous studies, we demonstrated that BCoV gains access to HRT-18 cells through membrane fusion as well as CME [7]. This process requires endosomal acidification, cathepsins, cholesterol, dynamin and microtubules. In addition, we demonstrated that Rab7 and Rab11 participate in BCoV entry into HRT-18 cells [7]. In this study, we present novel evidence demonstrating that BCoV enters PBIECs via multiple pathways, including direct membrane fusion, CME, CavME and macropinocytosis. This process is regulated by endosomal acidification, dynamin, microtubules, and cathepsin activity. Furthermore, efficient BCoV entry into PBIECs requires the participation of the endosomal trafficking proteins Rab5, Rab7, and Rab11. In short, these findings suggest that BCoV adopts cell type-specific entry strategies when infecting different permissive host cells.

An acidic environment is essential for viral endocytosis and endocytosis-mediated membrane fusion. A growing body of evidence indicates that viral entry into host cells requires acidic conditions, including Seneca Valley virus (SVV) entry into PK-15 cells [30], Newcastle disease virus (NDV) entry into chicken macrophages [14], and feline coronavirus entry into host cells [31]. Our previous work has also demonstrated that BCoV entry into HRT-18 cells is mediated by endosomal acidification [7]. In the present study, CQ and NH_4_Cl were used to inhibit endosomal acidification. Both treatments significantly inhibited BCoV entry into PBIECs, further underscoring the essential contribution of endosomal acidification to the BCoV life cycle. Coronaviruses typically initiate infection by fusing their lipid envelope with the membrane of the host cell. The S glycoprotein is essential for this process and is a major determinant of coronavirus tropism and pathogenicity. Structurally, the S protein ectodomain consists of two distinct subunits, S1 and S2; the S1 subunit mediates attachment to cellular receptors, whereas the S2 subunit drives membrane fusion between the virus and the host cell [8]. This fusion step can occur either at the plasma membrane or within acidic endosomal compartments. In this study, we employed the fusion inhibitor SSAA09E3, which directly blocks membrane fusion. Our results showed that SSAA09E3 significantly inhibited both BCoV entry into and infection in PBIECs. Several coronaviruses, such as SARS-CoV-2 and Middle East respiratory syndrome coronavirus (MERS-CoV), are known to trigger cell-cell fusion and syncytium formation [32]. IFA and flow cytometry further demonstrated that SSAA09E3 markedly suppressed BCoV-induced syncytium formation in PBIECs. Collectively, these findings indicate that BCoV entry into PBIECs relies on membrane fusion. Notably, cleavage and activation of the S protein by host-derived proteases are critical prerequisites for coronavirus-driven membrane fusion. Next, we used the cysteine protease inhibitor E64D (targeting cathepsins, including cathepsin L and cathepsin B) and the serine protease inhibitor camostat (targeting TMPRSS2) to determine which class of host proteases is required for BCoV entry into PBIECs via membrane fusion. Our results showed that treatment with E64D significantly inhibited BCoV entry into PBIECs, whereas camostat treatment had no effect, indicating that BCoV entry into PBIECs requires the involvement of cathepsins but is independent of TMPRSS2. These findings are consistent with our previous observations in HRT-18 cells, in which BCoV entry was shown to depend on cathepsins but not on TMPRSS2.

A critical stage in host cell entry for the majority of viruses is the hijacking of the cellular endocytic system to form intracellular vesicles and direct them to endosomes or other organelles, thereby initiating subsequent stages of infection [33]. To date, the major endocytic pathways exploited by viruses include CME, CavME, and macropinocytosis. Among these, the molecular mechanisms of CME have been most extensively characterized. CME involves the assembly of clathrin-adaptor protein complexes that induce plasma membrane invagination, followed by dynamin-mediated membrane scission to release endocytic vesicles into the cytoplasm [24]. In contrast, CavME relies on the assembly of caveolin and associated signaling molecules to form flask-shaped caveolae at the plasma membrane, serving as an alternative or complementary pathway to CME [34]. Macropinocytosis is characterized by actin cytoskeleton rearrangement-driven formation of large, nonselective vesicles [24]. Different viruses exhibit remarkable specificity and flexibility in their choice of endocytic entry pathways. For example, SARS-CoV-2 and channel catfish virus (CCV) enter host cells via CME [35,36], while African swine fever virus (ASFV) depends on both clathrin and dynamin for internalization [37]. Foot-and-mouth disease virus (FMDV) utilizes both CME and CavME to enter CHO-677 cells [38]. HCoV-OC43 enters HCT-8 cells via

CavME, a dynamin-dependent pathway [39], whereas canine respiratory coronavirus exploits CavME to infect HRT-18 G cells [39]. Viruses reported to utilize macropinocytosis include Ibaraki virus (IBAV) entering hamster lung (HmLu-1) cells and human immunodeficiency virus type 1 (HIV-1) entering macrophages [40,41]. In addition, porcine enteric alphacoronavirus (PEAV) enters Vero/IPI-2I cells through CME and CavME [42] as well as macropinocytosis; Orf virus (ORFV) enters goat lip epithelial cells and goat mammary fibroblasts via CME and macropinocytosis [43]. Bovine parainfluenza virus type 3 (BPIV3) gains entry into Madin-Darby Bovine Kidney (MDBK) via both CME and macropinocytosis [44], while its entry into HeLa cells relies exclusively on CME and dynamin [45]. Collectively, these findings indicate that viral entry pathways are highly virus-specific and strongly dependent on the cell type. In this study, PBIECs were treated with endocytic pathway inhibitors (CPZ, dynasore, nystatin, and blebbistatin) as well as siRNAs targeting key endocytic components (CLTC, DNM2, and CAV1). All of these treatments significantly inhibited BCoV entry. These findings demonstrate that BCoV entry into PBIECs relies on CME, CavME, macropinocytosis and requires dynamin. Notably, this entry mechanism differs from that observed in HRT-18 cells, where BCoV entry is dependent on dynamin and CME but independent of CavME and macropinocytosis. The utilization of distinct endocytic pathways by the same virus in different cell types has also been reported for other viruses. Such differences likely represent an adaptive strategy employed by viruses to enhance entry efficiency and ensure successful infection across diverse cellular microenvironments.

Viral entry often disrupts host cholesterol homeostasis to promote viral survival, while host cells attempt to limit viral entry by reducing cholesterol availability [46]. Treatment of N2a cells with MβCD, a cholesterol-depleting agent, inhibits rabies virus (RABV) attachment and fusion, thereby suppressing viral infection [47].

Cholesterol has also been shown to participate in the endocytic uptake of BPIV3 in HeLa cells, as well as in Japanese encephalitis virus entry into PK-15 cells [48]. In herpes simplex virus type 1 (HSV-1), removal of cellular cholesterol substantially diminishes virion fusion activity, as assessed “fusion-from-without” assays, highlighting the importance of cholesterol during the membrane fusion stage of HSV-1 entry [49]. Earlier work demonstrated that BCoV entry into HRT-18 cells depends on cholesterol [7]. Here, cholesterol depletion markedly inhibited BCoV infection; however, viral attachment and entry were not detectably affected. These results indicate that BCoV entry into PBIECs is cholesterol-independent, whereas viral infection requires cholesterol. This phenomenon is similar to that observed for influenza virus, in which cholesterol depletion does not affect viral entry but significantly impairs viral infection. Therefore, cholesterol is dispensable for BCoV entry into PBIECs.

Microtubules are core components of the cytoskeleton that function as tracks for vesicular and organelle transport, while also contributing to the maintenance of cell morphology, regulation of cell division, and organelle positioning [50]. Accumulating evidence indicates that viral entry and intracellular trafficking depend on the microtubule network. For example, Borna disease virus (BDV) requires microtubules during the early stages of entry, prior to early endosome formation [51]; respiratory syncytial virus (RSV) particles are transported along microtubules during entry, and disruption of microtubules impairs this transport [52]; and microtubules are involved in HIV-1 transcytosis, facilitating microtubule-dependent transfer of internalized virions to target cells [53]. Consistent with earlier reports showing that BCoV entry into HRT-18 cells depends on intact microtubules [7], in this study, disruption of microtubule polymerization by colchicine significantly inhibited BCoV entry, indicating that microtubules play an essential role in this process.

Rab proteins act as key regulatory switches in intracellular membrane trafficking and coordinate vesicle budding, directional transport, anchoring, and fusion, thereby ensuring the precise delivery of membrane-associated cargo to specific intracellular destinations [24]. Notably, many viruses exploit Rab protein-mediated trafficking pathways to facilitate their endocytosis and subsequent intracellular transport, effectively hijacking host cellular machinery to support their infection cycles [17]. In this context, viruses frequently exploit Rab5-positive early endosomes, Rab7-associated late endosomes, and Rab11-labeled recycling endosomes to facilitate endosomal trafficking during infection. Different viruses exploit distinct endosomal routes; for example, PHEV enters Neuro-2a cells via early and late endosomal pathways [18], JEV traffics through early and recycling endosomes in baby hamster kidney (BHK-21) cells [54], and Newcastle disease virus requires early endosomes for entry into chicken macrophages [14]. To delineate the intracellular routing of BCoV following endocytic uptake in PBIECs, siRNA-mediated knockdown of Rab5, Rab7, and Rab11 was performed. Suppression of any of these Rab proteins resulted in a marked reduction in BCoV entry into PBIECs. Consistently, confocal imaging demonstrated that incoming BCoV particles colocalized with Rab5-, Rab7-, and Rab11-enriched compartments during entry. These findings indicate that BCoV entry into PBIECs requires trafficking through early, late, as well as recycling endosomes. This trafficking pattern differs from our previous observations in HRT-18 cells, in which BCoV entry depended only on Rab7 and Rab11, but was independent of Rab5 [7]. Such cell type-specific disparities in endosomal trafficking have also been described for other viruses. For instance, efficient entry of CSFV into 3D4/21 cells is dependent on trafficking via Rab5, Rab7, and Rab11 [20], whereas entry into PK-15 cells relies solely on Rab5- and Rab7-mediated transport and is independent of Rab11 [55]. In summary, this work presents a comprehensive analysis of the cellular mechanisms governing BCoV entry into PBIECs. Our findings demonstrate that BCoV enters PBIECs via membrane fusion and three endocytic routes, including CME, CavME, and macropinocytosis. After internalization, BCoV traffics sequentially through early and late endosomes and subsequently exits through recycling endosomes. In addition, dynamin, an acidic environment, cathepsins, and microtubules are all required for efficient BCoV entry into PBIECs. Previous studies on BCoV have primarily relied on HRT-18 cells, a human-derived cell line. In the present study, we systematically characterized the BCoV entry mechanism using a physiologically relevant primary cell model. HRT-18 cells and PBIECs differ substantially in terms of endocytic regulation, receptor expression profiles, membrane lipid composition, and overall cellular physiology. In contrast, PBIECs, as the natural target cells of BCoV in cattle, more accurately recapitulate the in vivo infection microenvironment, including protease expression profiles, intact epithelial polarity, and native receptor distribution. Together with our previous work, the present findings demonstrate that BCoV employs distinct entry strategies in HRT-18 cells and PBIECs. Importantly, in addition to confirming the involvement of CME-mediated entry, our results further demonstrate that CavME and macropinocytosis contribute substantially to BCoV entry into PBIECs. These findings suggest that BCoV utilizes a more diverse entry strategy in its natural target cells than in immortalized cell lines, highlighting the importance of physiologically relevant primary cell models for investigating coronavirus entry mechanisms. This phenomenon is consistent with reports on multiple other viruses and further confirms that viral entry pathways exhibit pronounced cell type specificity. Such specificity is not a random event but rather reflects a finely tuned adaptive strategy shaped during long-term virus-host coevolution. Based on the distinct entry mechanisms identified in this study, we will focus on identifying the key host factors that regulate BCoV entry pathway selection in different cell types and elucidating how these factors influence pathway choice by modulating the expression of endocytic components or intracellular microenvironmental conditions. Together, these findings establish a mechanistic framework that may inform the development of broad-spectrum antiviral interventions aimed at cell type-specific viral entry mechanisms and key molecular determinants.

Nevertheless, several limitations of this study should be acknowledged. The PBIECs used in this study were isolated from a single healthy neonatal calf. Although all experiments were independently repeated in three separate experiments and yielded highly reproducible results, this inherent limitation means that our findings may not fully capture the inter-individual biological variability within the bovine population.

## Materials and methods

### Virus, cells, and plasmid

Isolation and preservation of BCoV (GenBank: OP866728.1) were conducted in our laboratory. PBIECs used in this study were isolated and purified in our laboratory from fresh intestinal tissue.

Isolation of PBIECs: cells were derived from intestinal tissues of a neonatal calf. Animal experimental procedures were reviewed and approved by the Animal Management and Animal Welfare Ethics Committee of Northwest A&F University (Ethical approval number: IACUC2025-0620). Animal experiments were carried out in June 2025. Specifically, one healthy 3-day-old Holstein bull calf was obtained from the Northwest A&F University (approval ID: SCXK (Shaanxi) 2024–010). Following humane euthanasia via intravenous administration of pentobarbital sodium (150 mg/kg), intestinal segments were collected and thoroughly washed with PBS containing penicillin-streptomycin-amphotericin B mixture (Sigma-Aldrich, Cat. A5955) prior to cell isolation. All experimental procedures were strictly conducted in accordance with the *Guideline for the Care and Use of Laboratory Animals* issued by the Ministry of Science and Technology of the People’s Republic of China and the ARRIVE guidelines. The tissues were then incubated in PBS containing 1 mmol/L dithiothreitol (DTT; Merck KGaA, Cat. 10,708,984,001) in a culture incubator for 5 min to remove surface mucus. Subsequently, the intestinal segments were digested in a mixed solution of type II collagenase (Gibco, Cat. 17,101,015) and neutral protease (Merck KGaA, Cat. 4,942,078,001) for 30 min. The digested intestinal epithelial layer was gently scraped using a sterile blade, and the digestion suspension was collected and centrifuged. After discarding the supernatant, the cell pellet was dispersed in DMEM/F12 medium (Gibco, Cat. 12,634,010) and filtered through a 400-mesh cell strainer (Beyotime, Cat. FSTR042). After centrifugation of the filtrate, the cell pellet was resuspended in culture medium containing 5% sorbitol (Sigma-Aldrich, Cat. S7547) and subjected to centrifugation at 800 rpm for 3 min. This washing step was repeated 5 times. Ultimately, the cells were cultured in medium supplemented with various growth factors and fetal bovine serum (FBS; Gibco, Grand Island, NY, USA), transferred to a cell culture incubator, and maintained under standard culture conditions. Cell attachment and morphology were monitored by microscopy.

Purification of PBIECs: PBIECs were further purified using a combination of differential adhesion and trypsinization to remove contaminating fibroblasts and other non-epithelial cells while minimizing the loss of intestinal epithelial cells, resulting in highly purified PBIECs.

Growth curve analysis of PBIECs: The viability of PBIECs was assessed from 1 to 7 d, and using the CellTiter-Glo® 3D Cell Viability Assay (Promega, Cat. G9681), 100 μL of detection reagent was dispensed into each well, followed by shaking and incubation at room temperature for 30 min to stabilize the luminescent signal. Luminescence was measured using a multifunctional microplate reader (Tecan, Switzerland) and expressed as relative fluorescence units (RLU). Four replicate wells were included for each time point. Cell growth curves were generated by plotting culture time (days) against the mean RFU values.

Plasmids: A recombinant expression plasmid encoding the BCoV nucleocapsid (N) protein was constructed based on the pEGFP-N1 vector. This plasmid served as a standard for the construction of a quantitative standard curve for the BCoV N gene, and the proliferation curve of BCoV in PBIECs was analyzed by absolute quantitative PCR.

### Cell viability assay for inhibitors

A cell viability assay was performed to determine the maximum non-cytotoxic concentrations of the inhibitors used in subsequent experiments. The following inhibitors and concentration ranges were tested: SSAA09E3 (membrane fusion inhibitor) (MedChemExpress, Cat. HY-138102), 5, 10, 20, and 40 μM; Dynasore (dynamin inhibitor) (TargetMol, Cat. T1848), 5, 10, 20, and 40 μM; CPZ (CME inhibitor) (Sigma, Cat. C0982), 5, 10, 20, and 40 μM; nystatin (CavME inhibitor) (Sigma, Cat. 475,914), 10, 20, 40, and 80 μM; blebbistatin (macropinocytosis inhibitor) (Sigma, Cat. 203,391), 5, 10, 20, and 40 μM; methyl-β-cyclodextrin (MβCD; cholesterol-depleting agent) (TargetMol, Cat. T4072), 0.63, 1.25, 2.5, and 5 mg/mL; colchicine (microtubule depolymerizing agent) (MedChemExpress, Cat. HY-16569), 200, 400, 600, and 800 nM; E64d (cathepsin activity inhibitor) (Selleck, Cat. S7393), 10, 20, 40, and 80 μM; and camostat mesylate (TMPRSS2 inhibitor) (MedChemExpress, Cat. HY-13512), 20, 40, 80, and 100 μM; Endosomal acidification inhibitors included chloroquine (CQ, Selleck, Cat. S6999), 60, 80, 100, 120 μM, and NH_4_Cl (Sigma, Cat. A9434), 5, 10, 20, 40 mM; Cholesterol (Thermo Fisher, Cat. A11470.18), 50, 80, 100, and 150 μM. PBIECs were plated in 96-well plates and exposed to the indicated concentrations of each inhibitor for 24 h. Cell viability was then assessed by adding CCK-8 reagent to each well, followed by incubation for an additional 3 h. The absorbance was recorded using a microplate reader to determine the maximum non-cytotoxic concentration.

### Immunofluorescence assay (IFA) and TCID_50_ assay

Following purification, PBIECs were processed for fixation in 4% paraformaldehyde and permeabilization in 0.1% Triton™ X-100. Cells were incubated overnight with a primary antibody against cytokeratin-18(CK-18; Proteintech, Cat. 10,830–1-AP; 1:400). Cells were then incubated with CoraLite488-conjugated goat anti-rabbit IgG (H+L) secondary antibody (CoraLite488 secondary antibody; Proteintech, Cat. SA00013-2;) at a dilution of 1:200. After a 10-min incubation with DAPI (Thermo Fisher, Cat. 62,248) at room temperature. The proportion of CK-18-positive PBIECs was examined using an inverted fluorescence microscope (Axio Observer, ZEISS, Germany).

When PBIECs in culture plates reached approximately 60% confluence, cells were treated with specific inhibitors or transfected with siRNAs targeting Rab5, Rab7, Rab11, CLTC, CAV1, or DNM2, followed by infection with BCoV. Culture supernatants were collected at 24, 48, and 72 h post-infection for TCID_5__0_ titration and IFA analysis. Primary antibody against BCoV N protein was used (1:100; laboratory-prepared).

TCID_50_ assay: The collected supernatant was diluted and inoculated into HRT-18 cells. After 48 h, cells were fixed, permeabilized, blocked, and then incubated with rabbit anti-BCoV N protein antibody. Cells were then incubated with CoraLite488 secondary antibody and stained with DAPI. Virus-positive wells were identified and recorded using an inverted fluorescence microscope. The TCID_50_ value was calculated.

### Confocal microscopy

Fluorescent labeling of BCoV: The lipophilic fluorescent dye DiD (Thermo Fisher Scientific, Cat. V22887, USA), which labels membrane structures, was used to stain the viral envelope of BCoV. The DiD dye was mixed with the virus at a dilution of 1:200. The tube was wrapped in aluminum foil to protect it from light and placed on a rotator for 2 h to allow DiD labeling. The unbound dye was removed by the Cytiva NAP-10 desalination column (GE Healthcare, Cytiva), and the DiD-labeled BCOV was obtained after filtration.

FITC-conjugated 70-kDa dextran was used as a specific marker of macropinocytosis in this study. PBIECs were divided into a control group and a blebbistatin-treated group. Cells in the treatment group were pretreated with blebbistatin at a final concentration of 10 μM for 24 h. FITC-labeled dextran was subsequently added to the culture medium to a final concentration of 5 mg/mL, followed by incubation at 37°C for 30 min. After incubation, the cells were washed three times with PBS to remove unbound dextran, fixed with 4% paraformaldehyde, and counterstained with DAPI. Fluorescence signals were then observed using a confocal laser scanning microscope.

Confocal imaging of DiD-labeled BCoV: The culture plates were coated with poly-L-lysine to enhance cell adhesion. 200 μL DiD-labeled BCoV was added to the coated plate and adsorbed at 4°C for 2 h. After fixation, it was incubated with BCoV N protein primary antibody, and then incubated with CoraLite488 secondary antibody. Coverslips were sealed using an antifade mounting medium (Thermo Fisher Scientific, Cat. P36980). Colocalization of DiD-labeled BCoV and BCoV N protein signals was examined using a laser scanning confocal microscope (LEICA TCS SP8), confirming efficient DiD labeling suitable for subsequent experiments.

Colocalization analysis of BCoV with endocytic markers: PBIECs were seeded onto coverslips in 24-well plates and cultured for 24 h. Cells were then infected with DiD-labeled BCoV for 1, 2, or 3 h, or with unlabeled BCoV for 24 or 48 h. After infection, cells were washed 3 times with cold PBS and incubated with DiD-labeled BCoV or antibodies against BCoV N protein in combination with antibodies against Rab7 (Sigma-Aldrich, Cat R8779-25 UL), Rab5 (Proteintech, Cat. 66,339–1-Ig), Rab11 (Proteintech, Cat. 67,902–1-Ig), clathrin heavy chain (CLTC; Proteintech, Cat. 66,339–1-Ig;), or caveolin-1 (CAV1, Proteintech, Cat. 16,447–1-AP), dynamin-2 (DNM2, Proteintech, Cat. 14,605–1-AP), FITC-labeled dextran (70-kDa, Beyotime, Cat. ST2947). Cells were subsequently incubated with CoraLite488-conjugated goat anti-mouse/rabbit IgG (H+L) (Catalog No. SA00013-1/-4). Nuclei were counterstained with DAPI for 10 min. Coverslips were sealed in antifade mounting medium and imaged via a laser scanning confocal microscope.

### Real-time quantitative PCR (RT-qPCR)

RNA was extracted using TRIzol reagent. RNA concentration was measured using a Thermo Fisher Scientific UV-visible spectrophotometer. Reverse-transcribed cDNA was used as a template for RT-qPCR analysis. The amplification protocol was as follows: initial denaturation at 95 °C for 30 s, followed by 40 cycles of denaturation at 95 °C for 5 s and annealing/extension at 60 °C for 30 s. Reactions were performed using a CFX Connect Real-Time PCR Detection System (Bio-Rad, USA). β-actin was used as the internal reference gene. Relative expression levels of the target genes (BCoV, Rab5, Rab7, Rab11) were calculated after normalization to β-actin using the 2^-ΔΔCt method. Primer sequences utilized for RT-qPCR analysis are summarized in Table 1. Both data visualization and statistical analyses were carried out with GraphPad Prism 8.

**Table 1.** Primers used for RT-qPCR.

Primer	Sequence (5’-3’)
β-actin-F	CACCGCAAATGCTTCTAGGC
β-actin-R	TGTCACCTTCACCGTTCCAG
BCoV-F	CGTTCTGGTAATGGCATCCTTA
BCoV -R	GTTTGCTTGGGTTGAGCTCTTCTA

### Western blot analysis

Cells were lysed on ice using RIPA buffer supplemented with 1% protease inhibitor cocktail. The supernatant obtained after centrifugation was combined with SDS-PAGE loading buffer, and protein samples were denatured before storage at −80°C.

Protein samples were separated by SDS-PAGE and transferred onto polyvinylidene difluoride (PVDF) membranes (Merck Millipore, Cat. ISEQ00010) using a wet transfer system. The following primary antibodies were used: anti-BCoV N protein, anti-β-actin (CST, Cat. 4967S), anti-Rab5, anti-Rab7, anti-Rab11, anti-CLTC, anti-CAV1 and anti-DNM2. All antibodies were diluted in accordance with the manufacturer’s recommended protocols. Membranes were then incubated with HRP-conjugated goat anti-mouse/rabbit IgG (H+L) (RS0001/RS0002) secondary antibodies at a dilution of 1:12,000. Protein bands were imaged with an Amersham ImageQuant 800 system (Cytiva, Sweden). Band intensities were quantified using ImageJ software, and data were analyzed and plotted using GraphPad Prism 8.

### siRNA design and transfection

siRNAs targeting the indicated genes were obtained from Tsingke Biotechnology Co., Ltd. Three distinct siRNAs were designed for each target gene, and the most efficient siRNA was selected for subsequent experiments based on silencing efficiency. The siRNA sequences are listed in Table 2. When PBIECs cultured in 6-well plates reached approximately 60% confluence, siRNA transfection mixtures were prepared using jetPRIME transfection reagent according to the manufacturer’s instructions.

**Table 2.** Sequences of siRNAs used in this study.

Target gene	Sense	Antisense
siRab5A-1	GGAUUAUCCUGAAGACAUA	UAUGUCUUCAGGAUAAUCC
siRab5A-2	GGCCGACCUAGCAAAUAAA	UUUAUUUGCUAGGUCGGCC
siRab5A-3	GGAAUCAGUGUUGUAGUAA	UUACUACAACACUGAUUCC
siRab7-1	GCUGCGUUCUGGUAUUUGA	UCAAAUACCAGAACGCAGC
siRab7-2	GAAACAAGAUUGACCUCGA	UCGAGGUCAAUCUUGUUUC
siRab7-3	CGGAAGUGGAGCUGUACAA	UUGUACAGCUCCACUUCCG
siRab11-1	GGAUGAACGUUGCUACUGA	UCAGUAGCAACGUUCAUCC
siRab11-2	GGGCAAUAAGAGUGAUCUA	UAGAUCACUCUUAUUGCCC
siRab11-3	GCAACAAUGUGGUUCCUAU	AUAGGAACCACAUUGUUGC
siCLTC-1	CAGAUGCUAUUCUAGGCAA	UUGCCUAGAAUAGCAUCUG
siCLTC-2	CGACAAAGUCUUCGAGAAA	UUUCUCGAAGACUUUGUCG
siCLTC-3	GGACAAGACUUCUCCAAGA	UCUUGGAGAAGUCUUGUCC
siCAV1-1	CGACGACGUGGUCAAGAUU	AAUCUUGACCACGUCGUCG
siCAV1-2	CAGUUGUACCAUGCAUUAA	UUAAUGCAUGGUACAACUG
siCAV1-3	GGAUCAAGACUUCUCCAAG	CUUGGAGAAGUCUUGAUCC
siDNM-1	GGACAAGACUUCUCCAAGA	UCUUGGAGAAGUCUUGUCC
siDNM-2	GAGCUAAUCAAUACAGUUA	UAACUGUAUUGAUUAGCUC
siDNM-3	GGAUCAAGACUUCUCCAAG	CUUGGAGAAGUCUUGAUCC

### Viral entry assays

Viral infection assay: PBIECs were transfected with siRNAs for 48 h, or pretreated for 24 h with the maximum working concentrations of chemical inhibitors. The cells were then infected with BCoV (MOI = 1). Cell lysates and culture supernatants were harvested at 24 h, 48 h, and 72 h post-infection and subjected to Western blotting, IFA, TCID_50_ and RT-qPCR analysis to determine BCoV N protein expression levels, numbers of infected cells, progeny virus titers, and viral genomic RNA copy numbers, respectively.

Virus attachment assay: PBIECs were treated with inhibitors or transfected with siRNAs following the same protocol used for viral infection assays. Cells were then infected with BCoV (MOI = 5) and incubated at 4 °C for 1 h to allow viral adsorption. Total cellular RNA was then isolated using TRIzol reagent after virus attachment and subjected to RT-qPCR analysis.

Virus entry assay: Following completion of the attachment assay, unbound virus particles were removed, and cells were transferred to 37 °C to allow viral internalization. Total RNA was collected at 1, 2, and 3 h following internalization, and subjected to RT-qPCR analysis.

### Flow cytometry and fluorescence microscopy analysis of BCoV-Induced syncytium formation

In this study, the lipophilic fluorescent dye DiD was used to label the plasma membrane, enabling both visualization and quantitative analysis of syncytium formation. The underlying principle is that DiD intercalate into the phospholipid bilayer of the cell membrane. When DiD-labeled target cells undergo membrane fusion to form multinucleated giant cells (syncytia), the redistribution of the dye results in an resulting in redistribution and altered fluorescence intensity. Flow cytometry was used to quantify the extent of membrane fusion and syncytium formation by measuring the mean fluorescence intensity (MFI). Cells were divided into six experimental groups [1]: blank control [2], SSAA09E3-only treatment [3], 3 h BCoV infection only [4], 3 h BCoV infection + SSAA09E3 [5], 24 h BCoV infection only, and [6] 24 h BCoV infection + SSAA09E3. After the indicated treatments, PBIECs membranes were labeled with DiD, and excess dye was removed. The resulting cell suspension was subjected to flow cytometric analysis on a BD high-speed cell sorter, with fluorescence detected in the APC channel. Post-acquisition data analysis was performed utilizing FlowJo software (v10).

To visualize the impact of SSAA09E3 on BCoV-induced syncytium formation, cells were examined using an inverted fluorescence microscope. PBIECs were assigned to three groups: a mock group without any treatment, a BCoV group infected with virus only, and an SSAA09E3+BCoV group treated with SSAA09E3 followed by viral infection. After all treatments, IFA was performed. Cells were probed with primary antibodies targeting BCoV N protein, and bright-field images, BCoV N protein staining, and nuclear staining were captured to assess syncytium formation.

### Fluorescent labeling and imaging of BCoV entry into PBIECs

BCoV particles were labeled with the lipophilic membrane dye DiD. The plasma membranes of PBIECs were labeled with 3,3’-dioctadecyloxacarbocyanine perchlorate (DiO; Cat. No. V22886, Thermo Fisher Scientific, USA). After staining for 12 min at room temperature in the dark, excess unbound dye was removed. DiD-labeled BCoV was added to DiO-labeled PBIECs at an MOI of 10 and incubated at 4°C for 1 h to allow stable viral attachment to the cell surface. Following incubation, real-time imaging was immediately performed for 5 min using a high-speed super-resolution confocal laser scanning microscope (LSM980, Carl Zeiss Microscopy GmbH, Germany) equipped with a 63× oil-immersion objective lens (NA = 1.4).

## Data analysis

Each experiment was conducted with a minimum of three independent replicates. GraphPad Prism 8.0 software was utilized for all statistical analyses, with data presented as mean ± SD (standard deviation). Statistical differences between groups were assessed via Student’s t-test or one-way ANOVA, with non-significant differences denoted by *p* > 0.05 and statistical significance was defined as follows: *p < 0.05; **p < 0.01; ***p < 0.001.

## Supplementary Material

QVIR-2026-0191.R1_OSB_DisclosureForm.docx

## Data Availability

The data that support the findings of this study are openly available in Figshare at https://doi.org/10.6084/m9.figshare.31375132, reference number [56].

## Open scholarship

This article has earned the Center for Open Science badge for Open Data. The data are openly accessible at https://doi.org/10.6084/m9.figshare.31375132
